# Substance use and associated factors among Gumuz people in Benishangul-Gumuz regional state, Mandura woreda, Northwest Ethiopia

**DOI:** 10.1186/s13011-019-0225-2

**Published:** 2019-09-03

**Authors:** Tesfa Gedif, Telake Azale, Adane Nigusie

**Affiliations:** 1 Public Health Officer, Metekel Zone Health Department, Benishangul-Gumuz Regional State Health Bureau, Asosa, Ethiopia; 2 Department of Health Education and Behavioral Science, Institute of Public Health, College of Medicine and Health Sciences, University of Gondar, 196 Gondar, Ethiopia

**Keywords:** Hazardous substances, Alcohol drinking, Northwest Ethiopia

## Abstract

**Background:**

Substance use related health and social problems are on the rise in sub-Saharan Africa. Currently, substance abuse is one of the most burning public health problems in Ethiopia. Although it has been known that this public health problem is an urgent issue, the real extent and magnitude of substance abuse is not yet properly explored. The purpose of this study was to assess substance use and associated factors among Gumuz people in Mandura Woreda, northwest Ethiopia.

**Methods:**

A community based cross sectional study was conducted involving 1588 adults, recruited using multi stage stratified simple random sampling technique. The data were collected at the household level by using pre tested Standardized questionnaire of Alcohol, Smoking and Substance Involvement Screening Test (ASSIST), Oslo social support scale and substance use questionnaire. The data was entered and cleaned using Epi Data version 3.1 and analyzed using Statistical Package for Social Science (SPSS) version 22 statistical package. Descriptive statistics and logistic regression were performed to examine the prevalence and predictors of substance use. An Adjusted odds ratio with 95% confidence interval was computed to determine the level of significance.

**Result:**

The overall life time substance use prevalence in the current study was 25.9% (95% CI = 23.7, 28.0). The three-month prevalence of Alcohol and tobacco use was 48.4 and 15.8% respectively. The three-month prevalence of hazardous alcohol and tobacco use were 25.8 and 7.8% respectively. Able to read and write (AOR = 3.79; 95%CI 2.34–6.15) in educational status and Strong social support (AOR = 0.39; 95%CI 0.27–0.58) were significantly associated with substance use.

**Conclusion:**

High level of substance use was detected in the current study setting. This high prevalence (three-month) of substance use affects the productive age groups which influence on the family and cause the rise of major public health and socio economic problems. The finding informed the need to integrate services for hazardous substance use such as brief intervention at different levels of primary care services in the district. Public health interventions to reduce hazardous substance especially on alcohol and smoking use also need to be initiate.

## Background

Substance use is a major public health concern that affects every level of society. Individuals, families, communities, and overall government spending are impacted by the use of licit and illicit substances. A 2015 study found that around 4.9% of the world’s adult population is believed to suffer from an alcohol use disorder [1]. Large epidemiological surveys have shown alcohol, tobacco (i.e., cigarettes), and marijuana has the highest prevalence rates across all age groups [2]. Alcohol is a serious public health problem. Globally, harmful use of alcohol results in the death of 2.5 million people annually. Alcohol contributes nearly to 4% of deaths to 6.2% of all male deaths related to alcohol compared to 1.1% death of females worldwide. Annually, 320,000 young people aged 15–29 years die from alcohol related causes resulting in 9% of all deaths in that age group globally [3].

Evidence from Center for Behavioral Health Statistics and Quality, National Survey on Drug Use and Health, shows that around one in four individuals among people ages 18 and older with serious mental illness has substance use habit [4]. Alcohol consumption and tobacco use are among the important risk factors for Non Communicable Diseases identified by the World Health Organization [5]. Substance use can contribute to the development of mental illness. Findings from the 2016 National Survey on Drug Use and Health show that 30.5% of respondents who have mental illness smokes cigarette in the past month, which is 66% higher than rate among those with no mental illness [6]. In addition, substance use can diminish the effectiveness of medications for physical condition and it was a serious public health problem that led to more than 42,000 deaths in 2016 alone [7, 8].

In the World harmful use of alcohol results in 3.3 million deaths each year, on average every person in the world aged 15 years or older drinks 6.2 l of pure alcohol per year. Less than half the population (38.3%) actually drinks alcohol, this means that those who do drink consumed on average 17 l of pure alcohol annually. At least 15.3 million persons have drug use disorders [9].

The harmful use of alcohol is one of the world’s leading health risks. It is a causal factor in more than 60 types of disease and injuries. The harmful use of alcohol is especially fatal for younger age groups and alcohol is the world’s leading risk factor for death among males age 15–59. Approximately 4.5% of the global burden of diseases and injury are attributable to alcohol. Alcohol consumption is estimated to cause 20 to 50% of cirrhosis of the liver, epilepsy, accidents, violence and cancer [9]. Recent trends indicate that the use of substances has dramatically increased, particularly in developing countries [10]. According to scientific evidence in Africa, an estimated 43% of those aged 15 years or above have ever used alcohol and 30% used it in the last year [11]. The global burden of disease attributable to alcohol and illicit drug accounts 5.4% of the total burden of disease [12]. The rapid economic, social, and cultural transitions that most countries in sub-Saharan Africa is now experiencing has created a favorable condition for increased and socially disruptive use of drugs and alcohol [13]. Substance misuse is a growing problem in Ethiopia, as in many developing countries. Alcohol and chat are the most frequent substances of abuse [14].

Studies in Ethiopia, Indicated that the prevalence of alcohol consumption has shown a significant increment [15]. A baseline survey in East gojjam zone revealed that, substance abuse such as high alcohol drinking, chat and Shisha were the push factors for early sex initiation to adolescents and youths [16].

According to the 2016 Demographic and Health Survey (DHS) report by the Central Statistics Authority (CSA) of Ethiopia, a national survey involving a representative sample from the age group 15–49 year old, 35% of women and 46% of men reported a history of alcohol consumption in their lifetime, 50% of women and 58% men of ever drinkers reported consumption of alcohol six or more days in the past month. Consumption rate was higher in urban areas, and the rates increased with age for both men and women [17].

Even if substance use has become a common problem in the study area, no information is available about the magnitude of substance use and factors contributing to its use. Therefore the aim of this study was to assess the magnitude of substance use and associated factors among Gumuz people in Mandura Woreda. The rationale behind this study is that, there are no data concerning commonly substance uses in Mandura district, though substance use is an emerging public health problem. And also, as far as our knowledge and searching effort, no study was conducted on substance use among Gumuz people. The problem is usually overlooked in the area. So, this study is designed to bridge the fore mentioned gaps.

## Methods

### Study design and setting

A community based cross sectional study was conducted in Mandura Woreda. The study was conducted from March to April 2018 in the Mandura Woreda, Metekel zone, Benishangul-Gumuz regional state. Mandura is located in the northwest 546 km from Addis Ababa (the capital city of Ethiopia) and 338 km from Asossa (capital city of Benishangul-Gumuz regional state) in the northern direction.

### Sample size

The sample size was calculated for both objectives and then we have taken the largest samples. Accordingly, sample size calculation for the first objective i.e. to determine the Prevalence of substance use was calculated using single population proportion formula;

$$ \mathrm{n}=\Big[{{{{}_{\mathrm{Z}\ \left(\upalpha /2\right)}}^2}_{\mathrm{p}\ \left(1-\mathrm{p}\right)/\mathrm{d}}}^2 $$

n = sample size

Z α/_2_ = 1.96 standard score corresponding to 95% CI

d = 0.05

*p* = 0.236 (Proportion of substance use) [18]

$$ \mathrm{n}={\left(\mathrm{Z}\upalpha /2\right)}^2\ \mathrm{p}\ \left(1-\mathrm{p}\right)/{\mathrm{d}}^2={(1.96)}^2\ 0.236\left(1-0/236\right)/{(0.05)}^2=\mathbf{258} $$

*n* = 258, since there is design effect we multiply by 2 then it will be 516 with 10% non-response rate, it will give 568.

Whereas the sample size calculation for the second objective to identify factors associated with substance use by using significant factors from previous study conducted on substance use among population of rural Ethiopia [15, 18] using EPI INFO STAT CALC with, 95% confidence interval, 5% margin of error, 2 design effects and adding 10% of non-response rate was 1588 which is larger than samples from single population proportion formula. Hence, the total final sample size was 1588 adults; age 18 and above years old.

### Sampling procedure

Multi stage stratified random sampling procedure was employed according to the setting (urban and rural). Then simple random sampling method was applied for selection of participants in each sub stratified population proportionally. First, from twenty three rural kebele (the lowest administrative unit in Ethiopia) and two urban kebele five and one respectively were randomly selected using simple random sampling technique. Next systematic sampling technique was employed to select households from each kebeles. The number of households sampled from selected kebeles was determined proportionate to population size.

Proportional allocation of the sample size was employed for each selected kebele based on the number of house hold they have.

There are a total of 3297 households who reside adults in the selected kebeles. The interval value (2nd) was calculated for selected kebeles by dividing the total households of each selected kebeles to the proportional sample size of the kebele. The initial household to be interviewed was selected randomly with lottery method and the next was selected every 2nd house. If more than one eligible adult was found in the same selected household, only one of them was chosen using lottery method for interview. And if no eligible candidate is identified in the selected household, the next household will be taken.

### Data collection tool and procedure

Data were collected using pre tested ASSIST (Alcohol, Smoking and Substance Involvement Screening Test), Oslo social support questionnaire and tobacco and alcohol use questionnaire. ASSIST is a brief screening questionnaire to find out about people’s use of psycho active substances. It was developed by the World Health Organization (WHO) and an international team of substance use researchers as a simple method of screening for hazardous, harmful, and dependent use of alcohol, tobacco and other psycho active substances [19]. The tools are WHO standard tool [20]. The data collectors were Diploma graduate nurses who collect the data by using interviewer administered questionnaire. The data collectors explain each question to the participant to help them understand the questions well and answer fill their own response. Three supervisors who is BSC degree graduate and familiar with the specific community were employed for smooth running of data collection process before and during data collection period. The principal investigators have followed and controlled overall data collection process, trained data collectors and supervisors, and performed pretest. The data was collected using interviewer-administered structured questionnaire which was prepared in English and then translated to local language (Amharic) which most communities could understand.

### Definition of study variables

The outcome variable of the study was substance use i.e. use of at least one of the substances (alcohol, khat, cigarettes, and illicit drugs) in an individual’s life time to alter mood or behavior.

The explanatory variable of interest of this study was socio-demographic (sex, age, educational status, marital status, occupation, and religion), environmental factors (availability and accessibility of hazardous substances /Alcohols, cigarettes or Tobaccos), inter personal factor (family, peer, Coworker/work mate) and Social support (Father, Mother, Brother, Sister, Relatives, Friends, Neighbors).

### Operational definition

**Substance use:** Use of at least one of the substances (alcohol, khat, cigarettes, and illicit drugs) in an individual’s life time to alter mood or behavior.

**Hazardous substance:** the two commonly used psychoactive substances; either both Alcohol and Cigarette smoking/tobacco use and one of them.

**Hazardous use:** is a pattern of psychoactive substance use which increases the risk of harmful consequences for the user.

**Hazardous substance use:** using psychoactive substances; alcohol and cigarette smoking/tobacco use in the past three months [21].

**ASSIST:** the Alcohol, Smoking and Substance Involvement Screening Test with sensitivity and specificity 97 and 90% respectively [22]. It is a brief screening questionnaire to find out about people’s use of psychoactive substances. The risk level of Tobacco use is, 0–3 low risk, 4–26 Moderate risk and 27 and above High risk. And the risk level of Alcohol use is; 0–10 low risk, 11–26 Moderate risk and 27 and above High risk [23].

**OSLO:** a three item social support questionnaire designed to measure perceptions of social support and satisfaction with that social support. Scale score = sum of individual scores; scores range from 3 to14. Score of 3–8 indicates ‘poor support’, 9–11 indicates ‘moderate support’ and 12–14 indicates ‘strong support’. Individual items can also be scored [24].

**Prevalence of substance use:** was measured by using WHO ASSIST V3 guideline. This was measured with “yes “or “no” item and about substance use in the past 3 month.

**Social supports:** were measured by using the Oslo – social support scale guide/questionnaires.

### Data processing and analysis

The collected data were entered and edited using Epi-Info version 7.00 software and then transferred to SPSS version 22 for further analysis. Specific substance involvement was calculated and categorized as Low, Moderate and High risk. Total social support calculated and categorized as poor and strong social support. Using descriptive methods, the data was summarized, prevalence of substance use was determined and odds ratios (OR) were obtained using logistic regression. The data obtained from individuals in each household are pooled to create a single large data set then the studies use the number of individuals substance use analyzed as the statistical n value, which is we assume the data gathered at each kebele to be an independent measurement so that we can use simple logistic regression by ignoring clustering [25]. The effect of Socio demographic (age, sex, education, marital status, occupation and Income) and psychosocial factors including social support on substance use was explored using crude and adjusted odds ratios. After checking the correlation of independent variables, significance was determined using crude and adjusted odds ratios with 95% confidence intervals. To determine the association between the different predictor variables with the dependent variable, first bi-variable analysis between each independent variable and outcome variable was investigated using a binary logistic regression model and then all variables having *p*-value < 0.2 in the bi-variable analysis were suggested as a criterion for variable selection for inclusion into a multivariable model. So that all variables with a *p*-value of < 0.2 in the bi- variable were analyzed for multi-variable logistic regression.

*P*– value < 0.05 with a 95% confidence interval were regarded as significant determinant factors and the strength of the association between the variables were classified based on their value of odds ratio (OR).

#### Data quality assurance

The questionnaire was pretested on communities of Dangila which has similar socio demographic characteristics with the study community.

The data collectors (supervisors) were trained, and proper instruction was given before the survey. The collected data were reviewed and checked for completeness before data entry.

### Ethical consideration

Ethical approval for the study was obtained from the institutionalized review board, institute of public health, university of Gondar. Official letter that explains the objectives of the study was written to the respected Metekel Zone administration and zonal health office. The zonal administration and zonal health office in turn were wrote a letter to study kebeles for cooperation respectively. The objectives and the benefits of the study were explained for the study subjects. Written consent was obtained from each participant. The right of the participants to withdraw from the study whenever they want to do so was respected. Anonymous questioner was used to protect the identity and confidentiality of the information obtains from individual participants.

## Results

### Socio demographic characteristics

Out of the total 1588 adults that participated in the survey, questionnaires from all respondents were complete and considered for analysis making the response rate 100%. Of the total 1588 respondents, 591 (37.2%) were age 45 and above years with a mean age of 44.7 (SD = ±3.17 years) (Table 1).

**Table 1:** Socio-demographic characteristics of participants in Benishangul-Gumuz regional state, Mandura Woreda, Northwest Ethiopia, 2018

Characteristics		Frequency	Percentage
Sex of respondents	Female	869	54.7
	Male	719	45.3
Age of respondents	18–24	173	10.9
	25–34	482	30.4
	35–44	342	21.5
	45 and above	591	37.2
Marital status of the respondents	Divorced	5	0.3
	Married	1525	96.1
	Single	51	3.2
	Widowed	6	0.4
Educational status of respondents	Un able read and write	863	54.3
	Able to read and write without formal education	500	31.5
	Primary	153	9.6
	Secondary	72	4.5
Religion	Catholic	613	38.6
	Muslim	308	19.4
	Orthodox	499	31.4
	Protestant	112	7.1
	Traditional	56	3.5
Occupation of the respondents	Farmer	709	44.6
	Gov/NGO	88	5.5
	House wife	725	45.7
	Merchant	66	4.2
Income monthly Pocket Money (Ethiopian Birr)	<=100	360	22.7
	100–299	594	37.4
	300–499	504	31.7
	500–999	115	7.2
	> = 1000	15	0.9

*Gov* Government employee, *NGO* Non-governmental organization employee

### Prevalence of substance use

The three-month prevalence of substance use among the respondents was 25.9% (95% CI = 23.7, 28.0). The overall three-month prevalence of alcohol use and tobacco use was 769(48.4%) and 251(15.8%) respectively (Table 2).

**Table 2:** Substance use characteristics of participants in Benishangul-Gumuz regional state, Mandura Woreda, Northwest Ethiopia 2018

Characteristics	Frequency	Percentage
Substance user		
Overall user	411	25.9
Male	271	63.9
Female	140	34.1
Alcohol user		
Overall user	769	48.4
Male	476	61.9
Female	293	38.1
Tobacco user		
Overall user	251	15.8
Male	158	62.9
Female	93	37.1
Hazardous tobacco user without alcohol		
Overall	2	0.1
Male	2	0.1
Social support		
Poor support	256	33.2
Strong support	515	66.8
Total	771	48.6
Tobacco users peer/family/		
Almost none	63	25.1
About half	114	45.1
Most	72	28.7
Total	251	15.8
Alcohol users peer/family/		
Almost none	126	16.4
About half	231	30.0
Most	412	53.6
Total	769	48.4
Sources of tobacco		
Shop/Market	31	28.18
Multiple source	79	71.82
Total	251	15.8
Source of alcohol		
Shop/Market	26	26.8
House, shop and Neighbor	410	53.3
Multiple source	333	42.3
Total	769	48.4

### Psycho-social factors

The social support of participants found to be 33.2% poor social support and 66.8% strong social support. The percentages of participants whose peer, family or work mate use tobacco were about 37.1% half and 49% most. The percentages of participants whose peer, family or work mate use alcohol were about 30% half and 53.6% Most. Nearly 72 % (71.82%) of the participants were found tobacco from multiple sources whereas 53.3% of participant’s sources of alcohol were shop, house and neighbors (Table 2).

### Factors associated with substance use

Initially different variables such as age, sex, amount of pocket money, religion, marital status, Educational status, Occupation and social support were considered for bivariate analysis. In the bivariate analysis the following variables showed a statistically significant association with substance use: sex, educational status, age, social support, age, and occupation. These variables and other variable with *p*-value < 0.2 at bivariate were taken and analyzed together using multivariate logistic regression model.

After controlling for the effects of potentially confounding variables using multivariate logistic regression model, Educational status, occupation, social support, and age were found to be statistically significant predictors of substance use. The odds of respondents whose educational level is secondary, Primary and able to read & write were 3.68(AOR = 3.68; 95% CI 1.83–8.22), 9.83(AOR = 9.83; 95% CI 4.37–22.1) and 3.79(AOR = 3.79; 95%CI 2.34–6.15) times more likely to substance use than those who were unable to read and write respectively. The odds of respondents who have strong social support were 61% less likely to substance use (AOR = 0.39; 95% CI 0.27–0.58) as compare to poor social support. Being a farmer and house wife has no statistically significant association with substance use as compare to merchant where as the odds of being government employee were 4.51(AOR = 4.51;95% CI 1.27–15.98) times more likely to use substance (Table 3).

**Table 3:** Factors associated with substance use among Gumuz people in Mandura Woreda, Benishangul-Gumuz, northwest Ethiopia, 2018

Variable	Substance use		Odds ratio(95%CI)	
		Yes (%)	Crude OR	Adjusted OR
Sex	Male	271 (56.7)	1.431 (1.07, 1.92)	1.48 (0.78, 2.83)
	Female	140 (47.8)	1.0	1.0
Education	Unable to read and write	201 (51.7)	1.0	1.0
	Read and write	144 (52.9)	1.05 (78, 1.44)	3.79 (2.34, 6.15)**
	Primary	35 (60.3)	1.42 (0.81, 2.5)	9.83 (4.37, 22.1)**
	Secondary	31 (59.6)	1.38 (0.77, 2.49)	3.68 (1.83, 8.22)**
Occupation	Farmer	262 (56.2)	0.61 (0.270, 1.373)	1.9 (0.74, 4.86)
	Government Employee	26 (81.3)	2.05 (0.624, 6.750)	4.51 (1.27, 15.98)*
	House wife	104 (42.4)	0.35 (0.15, 0.80)	0.67 (0.26, 1.74)
	Merchant	19 (67.9)	1.0	1.0
Social support	Poor support	186 (72.7)	1.0	1.0
	Strong support	225 (43.7)	0.29 (0.21, 0.40)	0.39 (0.27, 0.58)**
Age			1.04 (1.03, 1.05)	1.08 (1.06, 1.11)**

NB: * = *P* value < 0.05 ** = *P* value < 0.001 *OR* Odd ratio *CI* Confidence Interval

The Hosmer-Lemeshow goodness of fit test (0.791) was checked on the logistic model for appropriateness, to assess how good the model is fit (*P*-value > 0.05)

## Discussion

In this study, the overall prevalence of hazardous substance use was 25.9% in which the majority (25.8%) was hazardous alcohol users. This finding was higher than the study conducted in rural Southern Ethiopia (21%). The reason for this difference might be due to differences in the study setting, the study in Southern Ethiopia was conducted among rural area whereas the current study was both in rural and Urban. Another difference might be the tool used; study in Southern Ethiopia used FAST whereas the current study was done by using ASSIST [15].

The prevalence of alcohol drinking in the current study was 48.4% and higher in men (61.9%) than women (38.1%). Which is higher as compared to a study conducted in South Africa (SA); in which nine (9%) of the populations were engaged in risky drinking and similarly more men (17%) had hazardous drinking than women (2.9%) [26], which is also higher than study from Ethiopia [27, 28] and other African countries [29]. It is more than twofold higher than the result in Dire Dawa (19.6%) [30], significantly higher than the study in Addis Ababa; 26.5% [27] and a study from southern Brazil 43% [29]. This difference might be due to study area/setting difference in the accessibility of different fabricated alcohols and also easy access and availability of alcoholic beverages in Ethiopia. Drinking alcohol is a socially acceptable as well as a behavior learned from parents and older siblings.

This finding revealed that the overall three-month prevalence of hazardous tobacco use was 7.8%, which was lower than former adult smokers who smoke daily 19.9% in US [31], this difference might be the accessibility of fabricated tobacco in the US, slightly higher than the study in Dire Dawa (5.6%) [27] but more than 2 times higher than among adolescents in Addis Ababa (2.9%) [32]. Easily access and availability may be reasons for the higher rates in our study. The absence of legal provision to control the spread cigarette aggravates the use in the area. In the current study, we found that the prevalence of substance use was considerably higher in men (63.9%) than women (34.1%). Additionally, we found that males were 1.48 times more likely to be substance users as compared with women. In our narrative review, we found that the prevalence of alcohol dependence in Ethiopia was 1%. The prevalence was significantly higher in men (1.9%) than women (0.9%) [33].

The consequences of the higher substance use in men and lower substance use in women might be the possible reasons a significantly greater magnitude and risk of hazardous substance use as well as alcohol dependence among women than men [34, 35].

The finding in the current study showed that substance use found significantly associated with age, which was consistent with the study done in Easter Ethiopia [15]. Study done among adults showed that the age group 20–24 and 45–59 years more likely to use tobacco, respectively as compared to age group 15–19 years [36]. This might be to avoid or to forget their problem and anxiety, which may arise from economic stress. This increased hazardous substance use with age are becoming the major public health and socioeconomic problems worldwide [9, 10].

In this finding being a government employee was significantly associated with hazardous substance use as compare to merchants. Government employees who were living in rural areas might feel discomfort and suffered from stress which made them expose for alcohol. Study in Korea showed regarding occupation workers had a greater risk of alcohol drinking [37]. The possible reason for alcohol use in both of the study might be Stress related to their work.

The current study showed that participants who can read and write, attend primary school, attend secondary school and above was significantly associated hazardous substance use as compared to being unable to read and write. However, a study done in Korean on adults showed that senior high school and college graduates had a decreased risk of high risk alcohol drinking [37]. The current study was negatively consistent with studies conducted among Korean adults of senior high school and college graduates. The possible reason for this difference might be participants in the current study involved in different works and might be exposed to social interaction and stress, which exposed them to hazardous substance use, and might have a chance to be accessible to alcohol and tobacco.

The current study revealed that social support reduces hazardous substance use and solve major health and socioeconomic problems. The strong social support was significantly associated hazardous substance use. This study was consistent with studies conducted in Southern Ethiopia [15]. Having strong social support is a very powerful factor for influencing behavior especially in young people. Adolescents who affiliate with non substance user socials may be pressured to be non user of substances. The study has strength in that it involved adults from both urban and rural settings in and used larger sample size. The study has limitations such as the study does not attempt to see the presence of any association between health status of adults and substance use behavior. We also assumed completely independent observations and did not take into consideration our multi-staged sampling strategy during the analysis. This may bias our estimates of the standard errors and significance tests.”The sample size calculation for the study is expected based on the overall substance use magnitude, but it is calculated by using a study done on alcohol and tobacco. There might be recalled bias among participants forgetting the exact frequency of substance use.

## Conclusions

The overall three-month prevalence of substance use in the current study among adults is high. The most commonly used substance among the respondents was alcohol and tobacco. Educational status, Occupation, Social support and age were found to be independent predictors of substance use among adults. This high prevalence of substance use affects the productive age groups which influence on the family and cause the rise of major public health and socio economic problems. The finding informed the need to integrate services for substance use such as brief intervention at different levels of primary care services in the district. Community based public health interventions to reduce substance use and also need to be initiate.

## Data Availability

The datasets generated and/or analyzed during the current study are available at University of Gondar, College of medicine and Health Science, Institute of Public Health and Benshangul Gumuz regional state health bureau in hard and soft copy repository [www.UoG.edu.et]. In addition the data are available from the authors upon reasonable request and with permission of the principal investigators (Tesfa Gedif- E-mail tesfagedif12@gmail.com).

## Abbreviations

ASSIST: Alcohol, Smoking and Substance Involvement Screening Test
BSc: Bachelor of Science
MPH: Master of public health
NCD: Non communicable diseases
PhD: Doctor of philosophy Degree
